# Pre-akinete formation in *Zygnema* sp. from polar habitats is associated with metabolite re-arrangement

**DOI:** 10.1093/jxb/eraa123

**Published:** 2020-03-09

**Authors:** Erwann Arc, Martina Pichrtová, Ilse Kranner, Andreas Holzinger

**Affiliations:** 1 University of Innsbruck, Department of Botany, Innsbruck, Austria; 2 Charles University, Faculty of Science, Department of Botany, Prague, Czech Republic; 3 University of Osnabrück, Germany

**Keywords:** Abiotic stress, green algae, metabolomics, pre-akinete, streptophyte, *Zygnema*

## Abstract

In streptophytic green algae in the genus *Zygnema*, pre-akinete formation is considered a key survival strategy under extreme environmental conditions in alpine and polar regions. The transition from young, dividing cells to pre-akinetes is associated with morphological changes and the accumulation of storage products. Understanding the underlying metabolic changes could provide insights into survival strategies in polar habitats. Here, GC-MS-based metabolite profiling was used to study the metabolic signature associated with pre-akinete formation in *Zygnema* sp. from polar regions under laboratory conditions, induced by water and nutrient depletion, or collected in the field. Light microscopy and TEM revealed drastic changes in chloroplast morphology and ultrastructure, degradation of starch grains, and accumulation of lipid bodies in pre-akinetes. Accordingly, the metabolite profiles upon pre-akinete formation reflected a gradual shift in metabolic activity. Compared with young cells, pre-akinetes showed an overall reduction in primary metabolites such as amino acids and intermediates of the tricarboxylic acid (TCA) cycle, consistent with a lower metabolic turnover, while they accumulated lipids and oligosaccharides. Overall, the transition to the pre-akinete stage involves re-allocation of photosynthetically fixed energy into storage instead of growth, supporting survival of extreme environmental conditions.

## Teaser

Survival of extreme environments of *Zygnema* sp. is associated with pre-akinete formation accompanied by metabolic re-arrangement that involves re-allocation of photosynthetically fixed energy into storage instead of growth.

## Introduction

Zygnematophyceae are currently believed to be the closest relatives to land plants (e.g. Wodniok *et al.*, 2011; de Vries and Archibald, 2018). Streptophytic green algae in the genus *Zygnema* build up massive mats in shallow freshwater habitats in alpine and polar regions, where they are exposed to numerous abiotic stress factors (Holzinger *et al.*, 2009; Pichrtová et al., 2014*a*, 2016*b*). Several authors investigated mechanisms that confer stress tolerance in Zygnematophyceae, with a view to understanding the requirements for terrestrialization (de Vries *et al.*, 2018). In nature, young, highly vacuolated vegetative cells of *Zygnema* spp. are frequently found in spring time, when liquid water is available after snow melt (Pichrtová *et al.*, 2014*a*). Towards the end of the growing season, the cells stop dividing, accumulate storage products, and change their morphology as they mature and are transformed to pre-akinetes (Pichrtová *et al.*, 2014*b*; Herburger *et al.*, 2015). This transition can be regarded as a part of the vegetative life cycle, occurring as a result of the seasonal changes. Using a RuBisCO marker, a total of 12 genotypes of *Zygnema* sp. collected in Svalbard were identified, highlighting the variability within the genus, impacting on some morphological traits such as chloroplast shape and cell width. However, across these genotypes, zygospore formation was only very rarely found (Pichrtová *et al.*, 2018), so it appears that *Zygnema* spp. preferably invest in the formation of pre-akinetes rather than sexual reproduction to survive unfavourable polar conditions. Under experimental conditions, pre-akinete formation occurs during prolonged cultivation and can be induced by nitrogen starvation (Pichrtová *et al.*, 2014*b*). Pre-akinetes are more tolerant to abiotic stress factors such as osmotic stress (Kaplan *et al.*, 2013; Pichrtová *et al.*, 2014*a*), desiccation (Pichrtová *et al.*, 2014*b*), and freezing (Trumhová *et al.*, 2019), and their reduced transcriptome changes compared with those found in young cells were proposed to reflect the pre-akinetes’ pre-acclimation to desiccation (Rippin *et al.*, 2017). Therefore, pre-akinete formation is considered a key strategy for survival of *Zygnema* under extreme conditions (Pichrtová *et al.*, 2016*b*).

Compared with young cells of *Zygnema* sp., pre-akinetes had lower photosynthetic activity (Herburger *et al.*, 2015) and more negative osmotic potentials (ψ=− 1.67 MPa versus ψ=− 0.80 MPa; Kaplan *et al.*, 2013). Others showed that sucrose is a main osmolyte in *Zygnema* sp. (Hawes 1990; Rippin *et al.*, 2017). Pre-akinetes also accumulated lipid bodies (Herburger *et al.*, 2015; Pichrtová *et al.*, 2016*a*) in conjunction with an increase in total fatty acids and a change in fatty acid composition, whereby oleic (C18:1) and linoleic acid (C18:2) increased the most (Pichrtová *et al.*, 2016*a*). A recent UHPLC-qToF-MS (ultra-high performance liquid chromatography-quadrupole time-of-flight mass spectrometry)-based metabolomics analysis of three *Zygnema* spp. demonstrated that young cells and pre-akinetes as well as different strains can be clearly discriminated by their metabolite profiles, with a focus on secondary metabolites, including terpenoids and alkaloids, and lipid metabolism (Holzinger *et al.*, 2018). In addition, Rippin *et al.* (2019) showed that in a natural *Zygnema* sp. mat from Svalbard, the transcriptome and metabolome of the top and bottom layers of this mat differed significantly.

We hypothesized that the transition from young cells to pre-akinetes is associated with the re-allocation of major pathways of primary metabolism towards the accumulation of storage compounds. We investigated the metabolite profiles of young cells and pre-akinetes of *Zygnema* sp. from polar habitats using GC-MS-based metabolite profiling. We first conducted a lab study with young (17-day-old) *Zygnema* sp. cells (genotype ‘C’ previously used by Pichrtová *et al.*, 2014*b*), compared with 2 months old stationary phase cultures and pre-akinetes generated by culturing for 2 months under nitrogen depletion. This approach allowed us to test if the different morpho-physiological traits of these cells described in earlier studies (Pichrotová et al., 2014*b*; Herburger *et al.*, 2015) can be associated with different metabolite profiles. Secondly, we analysed young cells and pre-akinetes collected in the field (genotypes ‘V’ and ‘U’ according to Pichrtová *et al.*, 2018), aiming to characterize metabolic changes associated with the formation of pre-akinetes under natural conditions. Metabolites showing common variation under both lab and field conditions are likely to provide insights into the adaptive traits of pre-akinetes.

## Materials and methods

### Algal material

The Antarctic strain *Zygnema* sp. C (CCALA 880), a well characterized strain (Pichrtová et al., 2014*b*, 2016*a*; Holzinger *et al.*, 2018), was chosen for the lab experiment. We used cultures in three developmental stages: (i) young cultures 17 d after inoculation on standard Bold’s basal medium (BBM; Bischoff and Bold 1963); (ii) stationary phase cultures 2 months after inoculation on BBM; and (iii) fully developed pre-akinetes 2 months after inoculation on BBM without nitrogen to accelerate pre-akinete formation (Pichrtová et al., 2014*b*, 2016*a*). All cultures were grown on Petri dishes with 1.5% agar media at 16 °C under a light:dark regime of 16:8 h (35 µmol m^–2^ s^–1^), and five biological replicates (*n* = 5) were harvested and freeze-dried on the same day.

Young cells and pre-akinetes of *Zygnema* sp. were selected based on morphological criteria (such as bright green chloroplasts in young cells and the accumulation of storage compounds in pre-akinetes) in a field site near Longyearbyen (Svalbard, High Arctic). Subsamples of algal mats were collected on 8 August 2015 from two algal mats as close to each other as possible (10 m apart), because it was not possible to find pre-akinetes and young cells in the same mat. The sampling site is situated on a platform near a stone mound on a path leading to the Sverdruphamaren mountain 329 m above sea level. The main source of water was a melting snowfield located directly above the platform. Mat 1 grew in a small streamlet at the edge of the platform (78°13.153'N, 15°35.088'E, temperature 4.7 °C, conductivity 40 µS cm^–1^, pH 7.3). The bright green biomass from the submerged bottom layer consisted predominantly of young vegetative cells. In addition, samples from the top layer of this mat were collected. Mat 2 grew in a shallow pool (78°13.143'N, 15°35.28'E, temperature 3.5 °C, conductivity 60 µS cm^–1^, pH 7.7) and consisted of pre-akinetes with very thick cell walls. It was later established that the two mats were formed by two different genotypes; *Zygnema* sp. from mat 2 was referred to as genotype U and *Zygnema* sp. from mat 1 as genotype V in Pichrtová *et al.* (2018). Genotypes C and V are closely related within the same clade; genotype U is more distant according to *rbc*L phylogenetic analysis (Pichrtová *et al.*, 2018). Upon sample collection, contaminants, including cell debris and other biological materials, mainly mosses and the cyanobacterium *Scytonema* sp., were manually sorted out under a stereo microscope and five biological replicates (*n* = 5) were rapidly frozen in liquid nitrogen. The analysis of the filtered 16S and 18S rRNA gene sequences indicated a high relative abundance of *Z. circumcarinatum* (>85%) as described in Rippin *et al.* (2019). Samples were transported from Svalbard to Austria in a dry shipper (Voyageur 12, Air Liquide Medical GmbH, Düsseldorf, Germany) and then stored at –80 °C until freeze-drying (Zirbus vaco2, Zirbus technology GmbH, Bad Grund, Germany).

### Light and transmission electron microscopy

Light microscopy of laboratory-grown *Zygnema* sp. C was performed using a Zeiss Axiovert 200 M light microscope (Carl Zeiss AG, Oberkochen, Germany) equipped with a Zeiss Axiocam MRc5 camera. The *Zygnema* sp. field samples were observed in an Olympus BX53 microscope with an Olympus DP72 microscope digital camera. Sample preparation for TEM followed the protocol of Holzinger *et al.* (2009) as described by Rippin *et al.* (2019). Chemical fixation, ethanol dehydration, and embedding in modified Spurr’s resin (Low viscosity embedding kit, Science Services, Munich, Germany) were conducted immediately after sampling in Svalbard. The embedded material was transferred to the laboratory and ultrathin sections were prepared from the embedded material (Reichert Ultracut, Leica Microsystems, Wien, Austria), counterstained, and investigated with a Zeiss Libra 120 transmission electron microscope (Carl Zeiss AG, Oberkochen Germany) at 80 kV, and images were generated with a 2 k SSCCD camera (Albert Tröndle Restlichtverstärker Systeme, Moorenweis, Germany).

### Metabolite extraction and derivatization

Small aliquots (~15 mg) of freeze-dried biomass were transferred to 2 ml Safelock round-bottomed Eppendorf tubes with 5×3 mm agate beads, frozen in liquid nitrogen, and homogenized using a ball mill for 5 min at 20 s^–1^ (TissueLyser II, Qiagen, Düsseldorf, Germany). The samples were then re-suspended in 1 ml of frozen (–20 °C) water:acetonitrile:isopropanol (2:3:3) containing [^13^C_6_]sorbitol at 4 µg ml^–1^ (Campro Scientific GmbH, Berlin, Germany) and extracted for 10 min at 4 °C with continuous shaking at 1000 rpm (Compact Digital Micro plate shaker, Thermo Scientific, Waltham, MA, USA). Insoluble material was removed by centrifugation at 20 000 *g* for 5 min at 4 °C. Then, 25 µl from the supernatant was collected and dried for 3 h in a vacuum centrifuge (Savant SPD111V P2 SpeedVac kit, Thermo Scientific). A processed blank was prepared following the same steps as the samples to be used as a quality control.

Chemical derivatization and GC-MS metabolite profiling analysis were performed essentially as previously described (Fiehn *et al.*, 2008, Fiehn, 2016). Vacuum-dried samples were re-suspended in 10 μl of methoxyamine-hydrochloride (Supelco 33045-U, Sigma-Aldrich, St Louis, MO, USA) at 20 g l^–1^ in pyridine (270970; 25 ml, Sigma-Aldrich) and incubated at 28 °C for 90 min, with continuous shaking, in a thermomixer (Ditabis^®^ MHR 13, GML, Innsbruck, Austria). Then, 90 µl of *N*-methyl-*N*-trimethylsilyl-trifluoroacetamide (MSTFA, Aldrich 394866; 10×1 ml, Sigma-Aldrich) were added and the reaction continued for 30 min at 37 °C. After cooling, the content of each tube was transferred to a 2 ml clear glass auto-sampler vial with micro insert (Agilent, http://www.agilent.com) for injection. Samples were injected between 2 h and 24 h after derivatization.

### GC-MS analysis

A 1 µl aliquot of each sample was injected using a TriPlus RSH autosampler on a Trace 1300 gas chromatograph coupled to a TSQ8000 triple quadrupole mass spectrometer operated with the Xcalibur software (Thermo-Scientific). Before and after each injection, the 10 µl syringe was washed three times with 5 µl of hexane and three times with 5 µl of ethyl acetate. The injector was operated in splitless mode, opening the split vent after 4 min, with a constant flow of helium at 1 ml min^–1^ and at a constant injector temperature of 250 °C. The glass liner (23467, Restek, Bellefonte, PA, USA) was exchanged after 25 sample injections. A 30 m Rxi-5Sil MS column with 0.25 mm internal diameter and an additional 10 m integrated guard column was used (13623-127, Restek). The oven temperature was held at 70 °C for 7 min then ramped at 10 °C min^–1^ to 330 °C, and held constant for 7 min. The transfer line temperature between the gas chromatograph and mass spectrometer was set to 300 °C. Electron impact ionization was employed at 70 eV with an ion source temperature of 330 °C. After 9 min solvent delay, mass spectra were acquired in full scan mode from *m/z* 50 to 600 at five spectra per second. All samples and blanks were randomized, and a mixture of alkanes (C10–C20, C22, C24, C28, C32, and C36 at 2 mg l^–1^ in hexane) was injected in the middle of the queue for calibration of external retention indices.

### Data processing

Raw data files were analysed with the Automated Mass Spectral Deconvolution and Identification System (AMDIS v2.71, http://chemdata.nist.gov/mass-spc/amdis/) (Stein, 1999). Retention times were first converted to Kováts alkane-based retention indices (Kováts, 1958). A retention indices/mass spectra library built using commercial standards injected and analysed by the same method and the same instrument, complemented by the NIST v2.0 (2011), Golm (Kopka *et al.*, 2005), and Fiehn (Kind *et al.*, 2009) databases, was then used for identification of metabolites. The AMDIS algorithm was applied with ‘medium’ resolution, sensitivity and shape requirement, and a minimal signal to noise ratio set to 80. Identifications were completed by manual interrogation of the above-mentioned libraries using the NIST Mass Spectral Search Program (MS Search v2.0). Identifications were only considered valid given a match of the spectrum (match score >80 in AMDIS, or >800 in MS-search) and retention index (±3 U difference from the home-made library) with library data. The most prominent unidentified compounds are reported as unknowns with their retention index and a characteristic fragment. A specific fragment was selected for the relative quantification of each compound based on the data generated with AMDIS, and the corresponding peak areas were then determined at the expected compound retention times using the Xcalibur v2.2 processing software (Thermo Scientific) with the ‘genesis’ algorithm. All peak integrations were subsequently assessed using the Xcalibur Quan browser. Missing values were replaced with the manually integrated background level at the expected peak retention time. Relative values of metabolite contents were determined by normalizing the peak areas of each metabolite to that of the internal [^13^C_6_]sorbitol standard and to the sample dry weights (see Supplementary Tables S1 and S2 at *JXB* online).

### Statistical evaluation of the metabolomic data

Statistical evaluation of the data was performed with R (R Core Team, 2019) using the ellipse, gplots, and ggplot2 packages (Wickham, 2016). The data were log transformed and Pareto scaled prior to unsupervised principal component analysis (PCA) and univariate statistics (*t*-test and one-way ANOVA). *P*-values were submitted to Benjamini and Hochberg (1995) false discovery rate (FDR) correction. Metabolites were reported as differentially accumulated when the adjusted *P*-value was <0.01 with a fold change >2 as compared with young cells. Hierarchical clustering was also performed using R with Pearson correlation as distance and Ward’s agglomeration method for clustering (Ward, 1963).

## Results

### Morphological and ultrastructural changes associated with pre-akinete formation

**Fig. 1.**
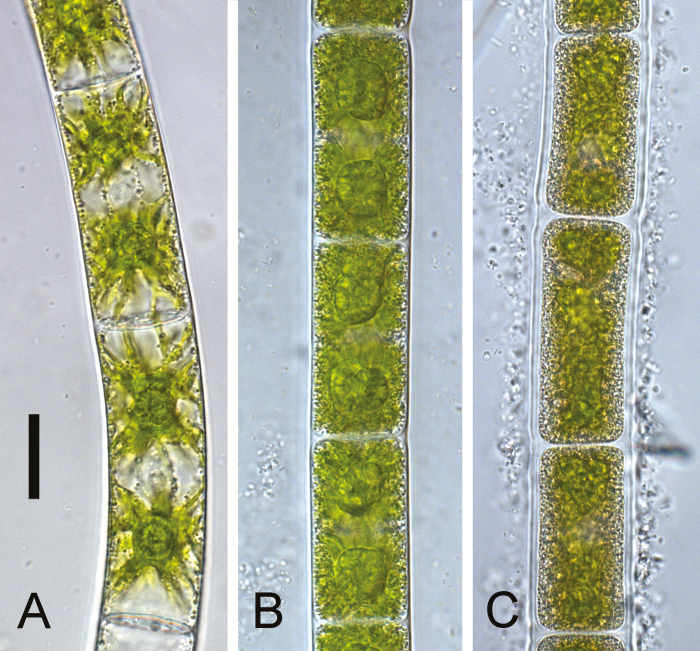
Morphology of *Zygnema* sp. used for the laboratory experiment. Light microscopy of (A) young cells; (B) stationary phase cells cultivated for 2 months; and (C) pre-akinetes cultivated for 2 months without nitrogen. Scale bar=20 µm.

In the laboratory study, three different culture stages were analysed, young cells (17 d old), stationary phase cells grown on standard medium (2 months old), and pre-akinetes cultivated on nitrogen-depleted medium (2 months old). Young cells of *Zygnema* sp. had large vacuoles and their bright green chloroplasts had distinct lobes, and nuclei were visible in the centre (Fig. 1A). The cytoplasm of the stationary phase cells was much denser than in the young cells. The individual chloroplast lobes were no longer discernible in stationary phase cells, and contained many vesicles and pyrenoids surrounded by a thick layer of starch (Fig. 1B). Pre-akinetes had a similar appearance to stationary phase cells, but the plastids were even more reduced and had lost their bright green colour, and the cell walls were thicker (Fig. 1C).

**Fig. 2.**
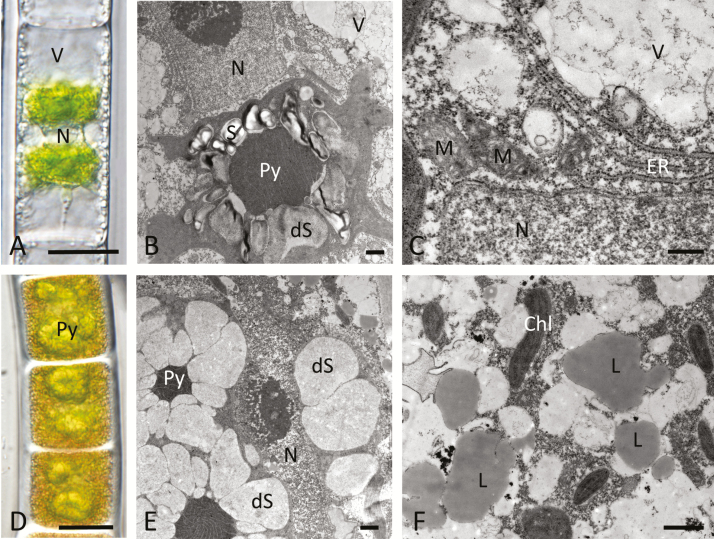
Young *Zygnema* sp. cells (A–C) and pre-akinetes from field samples in Svalbard. Light microscopy (A, D) and TEM (B, C, E, F) (A) of a young cell with a high degree of vacuolization; (B) overview of the central part of the cell with a pyrenoid surrounded by intact and partially degraded starch grains; (C) detail of (B) with the nucleus, mitochondria, and massive ER cisternae; (D) pre-akinetes filled with storage compounds and large pyrenoids; (E) pyrenoids surrounded by degraded starch grains; (F) accumulation of lipid bodies in the cytoplasm. Abbreviations: Chl, chloroplast; L, lipid body; M, mitochondrion; N, nucleus; Py, pyrenoid; ER, endoplasmatic reticulum; S, starch; dS, degraded starch. Scale bars: (A, D) 20 µm, (B, E, F) 1 µm, (C) 500 nm.

For the field study, bright green young *Zygnema* sp. cells isolated from the bottom layer of mat 1 were selected (Fig. 2A–C). Light microscopy showed that the cells had a diameter of ~25 µm with the nucleus in the centre, and two chloroplasts. The transparent cytoplasm in the periphery formed individual strands surrounding the large vacuoles (Fig. 2A). TEM confirmed the high degree of vacuolization and showed that the pyrenoids were surrounded by numerous starch grains. A few starch grains were degraded in young cells, and filamentous structures appeared (Fig. 2B). The central nucleus of young cells was surrounded by numerous endoplasmic reticulum (ER) cisternae and mitochondria, indicative of an active metabolism (Fig. 2C).

In contrast, pre-akinetes collected from mat 2 had a yellowish colour and a very dense cytoplasm that filled the entire cell from which chloroplasts were not clearly distinguishable apart from having prominent pyrenoids (Fig. 2D). The pre-akinetes had a diameter of ~33 µm. TEM confirmed the presence of numerous pyrenoids that appeared swollen. All starch grains were degraded, and the remaining electron-translucent areas showed filamentous structures (Fig. 2E). The cytoplasm contained numerous lipid bodies and individual chloroplast lobes (Fig. 2F).

### Metabolite re-arrangement associated with pre-akinete formation

**Fig. 3.**
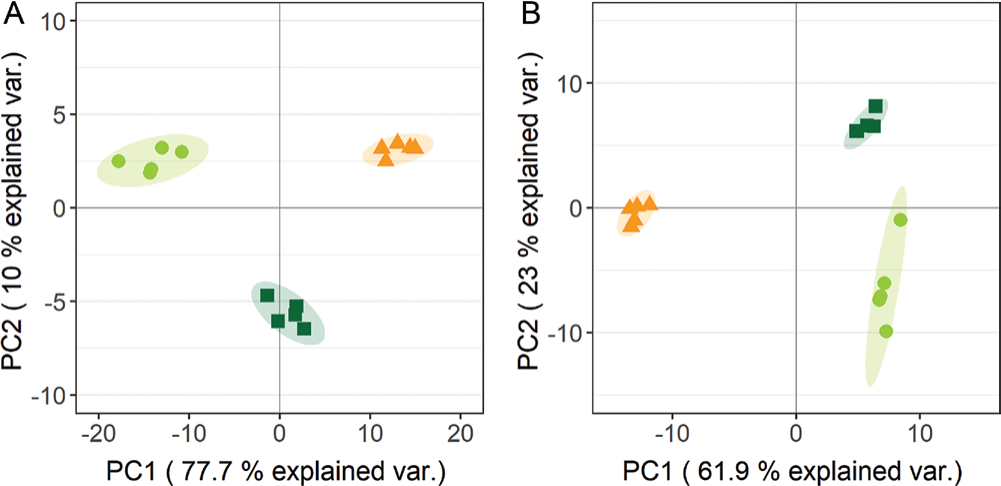
Comparison of metabolite profiles of *Zygnema* sp. (A) cultured in the laboratory and (B) collected in the field, by PCA. (A) Young cells (circles), 2 months old stationary phase cells (squares), and pre-akinetes (triangles). (B) Cells from the bottom (circles) and the top layer of mat 1 (squares), and pre-akinetes from mat 2 (triangles).

In *Zygnema* sp. grown in the laboratory, 146 compounds were detected, 109 of which were identified (Supplementary Table S1). PCA of all metabolites showed that the first principal component (PC1) separated young cells, stationary phase cells, and pre-akinetes, accounting for 77.7% of the variation (Fig. 3A), and the position of the stationary phase cells suggests that they represent an intermediate stage between young cells and pre-akinetes. The second principal component (PC2) separated stationary phase cells from young cells and pre-akinetes, accounting for 10% of the variation (Fig. 3A).

In *Zygnema* sp. collected from the field, 169 compounds were detected, 114 of which were identified (Supplementary Table S2). In the PCA, PC1 separated the young cells from the bottom and top layer of mat 1 from the pre-akinetes of mat 2, accounting for 61.9% of variation; PC2 separated the cells from the top and the bottom layer of mat 1, accounting for 23% of the variation (Fig. 3B).

**Fig. 4.**
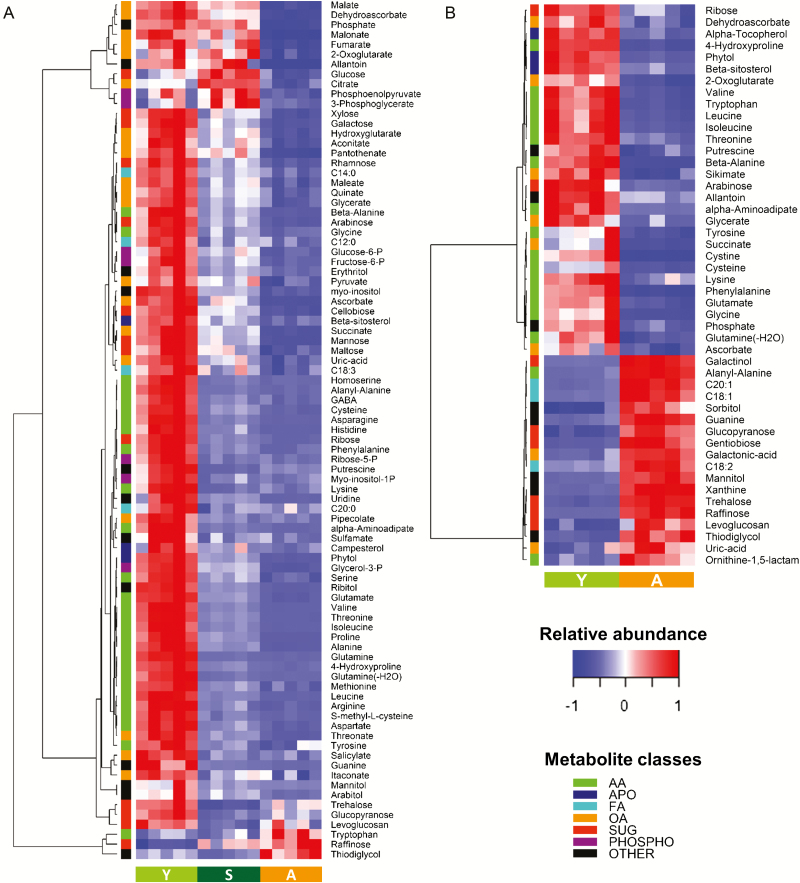
Differences in metabolite contents between *Zygnema* sp. cells at different stages from young cells to pre-akinetes under laboratory and field conditions. (A) Metabolites differentially accumulated between young cells (Y), stationary culture (S), and pre-akinetes (A) grown in the laboratory (one-way ANOVA *P*-value <0.01 after FDR correction, max log_2_ ratio >1). (B) Differences in metabolite contents between field-collected young cells and pre-akinetes (*t*-test *P*-value <0.01 after FDR correction, log_2_ ratio >1). Abbreviations: AA, amino acids (and associated metabolites); APO, apolar compounds; FA, fatty acids; OA, organic acids; SUG, sugars; PHOSPHO, phosphorylated compounds.

In cells cultured in the lab, 85 identified metabolites were differentially accumulated. Compared with young cells, most metabolites were down-accumulated in stationary culture cells and pre-akinetes (Fig. 4A). The contents of most amino acids and associated metabolites were strongly reduced in both stationary cultures and pre-akinetes as compared with young cells, whereby the highest variations were observed for glutamine, arginine, and histidine. A few molecules associated with nucleic acid metabolism, such as ribose-5-phosphate, guanine, and uridine, the sugar alcohols ribitol, mannitol, and arabitol, and apolar molecules such as phytol and campesterol showed a similar pattern of accumulation. In contrast, intermediates from the glycolysis pathway, including the hexose phosphates fructose-6-phosphate and glucose-6-phosphate, glucose, 3-phosphoglycerate, phosphoenolpyruvate, and pyruvate remained at higher levels in stationary culture cells as compared with pre-akinetes. Similar variations were found for key intermediates of the tricarboxylic acid (TCA) cycle including citrate, aconitate, 2-oxoglutarate, succinate, fumarate, and malate, the contents of which in stationary culture cells were often comparable with those of young cells, or higher in the case of citrate. The contents of several monosaccharides, including pentoses (xylose and arabinose) and hexoses (galactose, mannose, and rhamnose), and the disaccharides maltose and cellobiose also decreased in between the three stages, from young cells to pre-akinetes. Conversely, the only metabolites which were up-accumulated in the nitrogen-depleted cultures were the amino acid tryptophan, and the trisaccharide raffinose (Fig. 4A). Pre-akinete formation was also associated with the accumulation of several molecules, which could not be identified by comparison with available libraries (Supplementary Table S1). Sucrose was one of the most abundant sugars, but only showed a slight increase during pre-akinete formation (<20% increase, Supplementary Table S1).

In the field study, 48 metabolites were differentially accumulated between the young *Zygnema* sp. cells (bottom of mat 1) and the pre-akinete cells (*Zygnema* sp., mat 2). Thirty of these compounds showed reduced accumulation, including most amino acids and a few associated biosynthesis intermediates such as shikimate (shikimate pathway) and α-aminoadipate (lysine biosynthesis from 2-oxoglutarate), as illustrated in Fig. 4B. As compared with young cells, pre-akinetes also contained fewer of the TCA cycle intermediates 2-oxoglutarate and succinate, the organic acids ascorbic and dehydroascorbic acid, the pentoses ribose and arabinose, and apolar molecules such as phytol, α-tocopherol, and β-sitosterol. In contrast, 18 compounds were up-accumulated in pre-akinetes including oligosaccharides such as gentiobiose, trehalose, and raffinose, the sugar alcohols galactinol, sorbitol, and mannitol, and free unsaturated fatty acids (oleic linoleic and eicosanoic acids). As for the laboratory study, sucrose was one of the most abundant sugars and showed a slightly higher contents in pre-akinetes (Supplementary Table S2).

### Common variation between the laboratory and field study

**Fig. 5.**
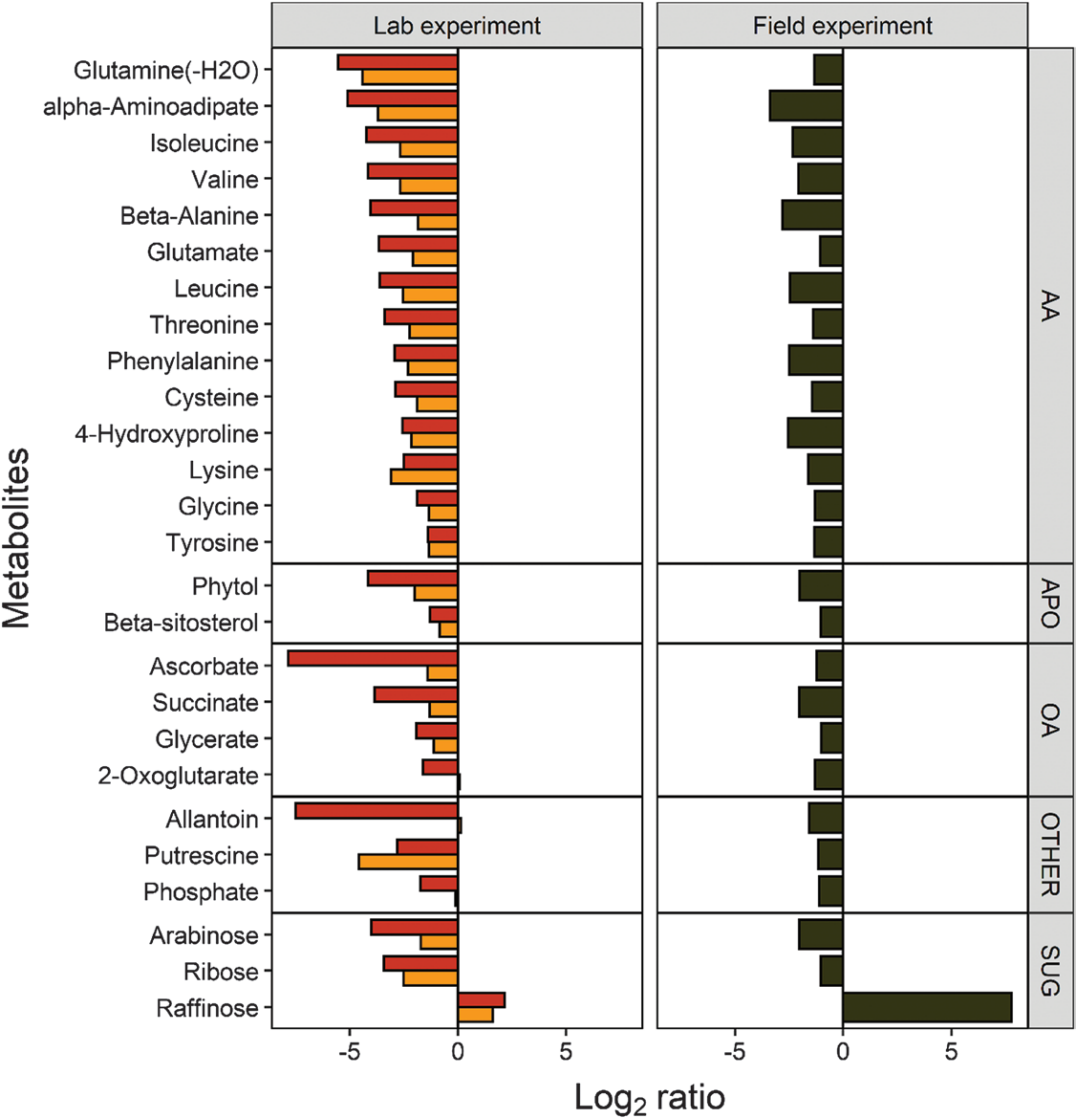
Metabolites showing either a positive or negative differential accumulation between pre-akinetes and young cells (FDR adjusted *t*-test, *P*-value <0.01, fold change >2) in both the field (right) and laboratory samples (left) are shown. Log_2_ ratios of laboratory-grown stationary phase cells versus young cells (orange bars) and pre-akinetes versus young cells (red bars), and field-collected pre-akinetes versus young cells (black bars) are shown.

In order to compare the laboratory and field study, the 27 metabolites showing common variations in both were selected (Fig. 5). Twenty-six metabolites were down-accumulated in pre-akinetes obtained in the lab and in the field study, including 14 amino acids and related compounds, the TCA cycle intermediates succinate and 2-oxo-glutarate, and ascorbate and dehydroascorbate. Raffinose was the only metabolite commonly up-accumulated in pre-akinetes.

## Discussion

In the present study, GC-MS-based metabolite profiling was used to study the metabolic signature associated with the transition from young cells into pre-akinetes in *Zygnema* sp. from polar regions. Pre-akinetes were either produced under laboratory conditions (Fig. 1C) or collected in the field (Fig. 2D). In the lab, pre-akinete formation can be induced in response to nutrient depletion by culturing *Zygnema* sp. on gradually drying agar for prolonged periods of time (e.g. 6–15 months) without providing fresh medium (Herburger *et al.*, 2015; Rippin *et al.*, 2017). This process can be accelerated by using nitrogen-free media, leading to pre-akinete formation within 2 months (Pichrtová et al., 2014*b*, 2016*a*), morphologically resembling 9 months old *Zygnema* sp. cells (Herburger *et al.*, 2015). In contrast, 2 months cultivation on standard BBM leads to an intermediate type of cells, here called stationary phase cultures. Young cells and pre-akinetes of *Zygnema* sp. were also collected from the field. Pre-akinetes produced by nitrogen starvation and those collected in the field had very similar morphology; cells were densely filled with storage compounds and had massive pyrenoids. The large vacuoles and the two chloroplasts observed in young cells were no longer clearly discernible in pre-akinetes, lipid bodies accumulated, and starch grains in the pyrenoids were degraded (Figs 1, 2), consistent with previous observations (e.g. McLean and Pessoney 1971; Holzinger *et al.*, 2009; Pichrtová et al., 2014*b*, 2016*a*; Herburger *et al.*, 2015). The morphology of the stationary phase cultures represented an intermediate state in between young cells and pre-akinetes (Fig. 1B). These changes in morphology in conjunction with reserve accumulation suggests a shift from metabolically highly active young cells towards stress-acclimated pre-akinetes. We first studied pre-akinete formation under controlled conditions in the lab, to provide a basis for the interpretation of metabolic differences between young cells and pre-akinetes under natural polar conditions, revealing that 27 differentially accumulated compounds showed common variation in both experiments.

### Pre-akinete formation under controlled conditions

The metabolite profiles of young cells, stationary cultures, and pre-akinetes reflected a gradual shift in metabolic activity (Fig. 3A). Compared with pre-akinetes, young cells had higher contents of organic acids, sugars, phosphorylated sugars, sugar alcohols, and amino acids, consistent with a higher metabolic turnover (Fig. 4A; Suplementary Table S1). Pre-akinetes showed an overall reduction in primary metabolites, with the exception of raffinose, gentiobiose, tryptophan, and at least seven unidentified compounds (Supplementary Table S1), which showed increased up-accumulation. Together with other osmolytes, the accumulation of raffinose could have contributed to the more negative osmotic potential observed in pre-akinetes compared with young cells (Kaplan *et al.*, 2013; Pichrtová *et al.*, 2014*b*). The stationary phase cells showed an intermediate profile that revealed a switch towards the metabolism of pre-akinetes. While maintaining turnover through glycolysis and the TCA cycle, accumulation of amino acids was strongly reduced, possibly indicative of reduced protein turnover, diverting energy into storage lipid synthesis (Fig. 4A), supported by an increase in total fatty acids (including bound fatty acids) and the accumulation of lipid bodies described by Pichrtová et al. (2016*a*). Most of the free fatty acids found by the metabolite profiling method did not show pronounced differences between young cells and pre-akinetes, suggesting that they were readily incorporated into storage lipids once synthesized.

### Pre-akinete formation under natural conditions

In a previous report, differences in the metabolite profiles of *Zygnema* sp. cells collected from the top and bottom layers of mat 1 were reported together with the results of a metatranscriptomic approach; the latter showed that the mat was dominated by *Zygnema* sp. (>85% relative abundance), with minor contamination by Bryophyta, Anthocerotophyta, cyanobacteria, and other organisms such as the mosquito *Aedes* sp. (Rippin *et al.*, 2019). It was demonstrated that the cells in the top layer had a higher metabolic turnover in response to higher levels of abiotic stress factors such as excessive irradiation, by up-regulating genes involved in photosynthesis, DNA repair, the cell wall, and carbohydrate, amino acid, and redox metabolism. Up-regulation of starch-degrading enzymes was linked to starch degradation, supported by ultrastructural observations, and higher maltose levels. It was proposed that the top layer shields the bottom layers from abiotic stress factors. The bottom layer comprised almost exclusively freshly divided cells with two stellate chloroplasts containing large vacuoles, whereas individual filaments in the top layer were pale and contained more storage compounds.

In the field, environmental and genotypic effects cannot be controlled, but environmental effects were minimized by collecting the samples at the same time of day and on the same day, and from mats that were as close to each other as possible. We aimed to select young cells and pre-akinetes based on morphological criteria, but we could not find mats containing both cell types during our sampling campaign. Therefore, we used the young cells from the bottom layer of mat 1 described in Rippin *et al.* (2019) and compared them with pre-akinetes found in mat 2. Data from the top layer (Rippin *et al.*, 2019) of mat 1 are included as a reference in the PCA (Fig. 3B). Both top and bottom layer cells from mat 1 had different metabolite profiles compared with pre-akinetes from mat 2, as shown by the clear separation along PC1. Top and bottom layer cells were only separated by PC2, showing that their metabolite profiles were more similar than those of the pre-akinetes.

In agreement with the starch degradation and the formation of lipid bodies observed by TEM (Fig. 2), mannose and free fatty acids (C18:1, C18:2, and C20:1), respectively, showed increased accumulation in pre-akinetes (Fig. 4B; Supplementary Table S2). The total fatty acid composition (including fatty acids stored in triacylglycerols) was previously investigated, showing that C18:1 and C18:2 in particular increased up to 17-fold (Pichrtová *et al.*, 2016*a*). Accumulation of lipid reserves is also important for other algal communities thriving in polar regions to survive upcoming harsh environmental conditions such as those dominated by *Chloromonas* sp. (Lutz *et al.*, 2015). In accordance with the large number of ER cisternae (Fig. 2) found in young cells but not in pre-akinetes, amino acids were down-accumulated in pre-akinetes, indicative of reduced protein turnover (Fig. 4B). In addition, amino acid synthesis was down-regulated in pre-akinetes. For example, lysine showed down-accumulation together with its precursors α-aminoadipate and 2-oxoglutarate (Fig. 4B). Furthermore, shikimic acid, tryptophan, tyrosine, and phenylalanine, which are key metabolites of the shikimate pathway, also showed down-accumulation in pre-akinetes. Genes encoding shikimate biosynthetic enzymes are highly conserved in the green lineage, and an estimated 30% of all fixed carbon is directed through the shikimate pathway. The shikimate pathway is key for the production of tryptophan, and tyrosine and phenylalanine are also produced through this cycle (Tohge *et al.*, 2013). In contrast to the lab study, we did not find strong evidence that glycolysis and the TCA cycle were down-regulated, although some of the TCA cycle intermediates were decreased in pre-akinetes (Fig. 4B). Of the antioxidants captured by the untargeted GC-MS-based metabolite profiling, α-tocopherol and ascorbate were decreased in pre-akinetes together with the tocopherol precursor, phytol (Gutbrod *et al.*, 2019), and dehydroascorbate, which is a product of ascorbate oxidation. Apart from its role as an antioxidant, ascorbate also stimulates the cell cycle (Horemans *et al.*, 2000), which is undesired in resting stages such as pre-akinetes. In contrast, several sugars and sugar alcohols were increased (mannitol, sorbitol, galactinol, raffinose, trehalose, and gentiobiose), enhancing the pre-akinetes’ tolerance of osmotic stress (Holzinger and Karsten, 2013).

### Metabolic re-arrangement associated with pre-akinete formation

The differences in the metabolite profiles of cells from the bottom layer of mat 1 and the pre-akinetes from mat 2 (Figs 3B, 4B) were probably caused by a combination of developmental, environmental, and genotypic effects. Therefore, we finally identified accumulation patterns common to the lab and the field study (Fig. 5) to elucidate which metabolic changes were characteristic for developmental effects, namely the transition from young cells to pre-akinetes. As per the statistical criteria applied, a total of 26 metabolites were decreased in both studies, and raffinose was increased (Fig. 5). Raffinose family oligosaccharides also increased upon desiccation stress in the streptophyte *Klebsormidium crenulatum* (Holzinger *et al.*, 2014), and are generally important osmolytes (Sengupta *et al.*, 2015). The most apparent common denominator of both studies was the decrease in accumulation of amino acids, and the TCA cycle was also down-regulated, in the lab study especially, as well as β-sitosterol, a membrane component (Deng *et al.*, 2016). Furthermore, the drastic changes in chloroplast morphology and ultrastructure (Figs 1, 2) are consistent with previous findings that pre-akinetes contained only about half of the chlorophyll contents of young cells (Holzinger *et al.*, 2018) and had reduced photosynthetic activity (Herburger *et al.*, 2015). This is further supported by the lower contents of phytol (Fig. 5), a key metabolite in chlorophyll synthesis and breakdown, in pre-akinetes in both studies (Gutbrod *et al.*, 2019).

In summary, pre-akinetes accumulated lipid reserves and osmolytes, whereas the cell cycle and metabolic activity were down-regulated, probably in preparation for surviving harsh environmental conditions. Therefore, the transition to the pre-akinete stage involves re-allocation of photosynthetically fixed energy into storage instead of growth, as also reported for starved cultures of other microalgae (Solovchenko, 2012; Vítová *et al.*, 2014). This ability to adjust metabolite composition in order to enhance the stress tolerance of pre-akinetes could also have supported terrestrialization of the Zygnematophyceae. In the future, it will be interesting to expand this study analysing changes on the proteome level, also including targeted analyses of secondary metabolites involved in stress response, which was outside the remits of this study.

## Supplementary data

Supplementary data are available at *JXB* online.

**Table S1.** Complete metabolite profiling dataset for the laboratory experiment.

**Table S2.** Complete metabolite profiling dataset for the field experiment.

eraa123_suppl_Supplementary_Table_S1

eraa123_suppl_Supplementary_Table_S2

## Abbreviations

BBM: Bold’s basal bedium
FDR: false discovery rate
PCA: principal component analysis
TCA: tricarboxylic acid
